# Supplementary material for: A Cationic Zn-Phthalocyanine Turns Alzheimer’s Amyloid β Aggregates into Non-Toxic Oligomers and Inhibits Neurotoxicity in Culture
Source: Int J Mol Sci. 2024 Aug 16;25(16):8931. doi: 10.3390/ijms25168931 (PMC11354870; doi:10.3390/ijms25168931)
Supplement: Supplementary file 1 [file ijms-25-08931-s001.zip › Supplemental figure legends-A beta cZnPc paper.pdf]

Supplemental figure legends.

Supplemental Figure S1.

A docking simulation was done to understand the interaction of $A\beta_{1-42}$ and cZnPc. In (a), the secondary structure of $A\beta_{1-42}$ monomer is shown. In (b), the docking view of $A\beta_{1-42}$ monomer and cZnPc is depicted. In (c), the hydrogen bonding with the amino acids of $A\beta_{1-42}$ monomer and cZnPc are shown. The positions of are mentioned beside the 3-letter symbol of amino acids. In (d), the 3-D docking view of $A\beta_{1-42}$ monomer and cZnPc is depicted.

Supplemental Figure S2.

A docking simulation was done to understand the interaction of $A\beta_{1-42}$ and cZnPc. In (a), the secondary structure of $A\beta_{1-42}$ 10-mer is shown. In (b), the docking view of $A\beta_{1-42}$ 10-mer and cZnPc is depicted. In (c), the hydrogen bonding with the amino acids of $A\beta_{1-42}$ 10-mer and cZnPc are shown. The positions of are mentioned beside the 3-letter symbol of amino acids. In (d), the 3-D docking view of $A\beta_{1-42}$ 10-mer and cZnPc is depicted.

Supplemental Figure S3.

A docking simulation was done to understand the interaction of $A\beta_{1-42}$ and cZnPc. In (a), the secondary structure of $A\beta_{1-42}$ 30-mer is shown. In (b), the docking view of $A\beta_{1-42}$ 30mer and cZnPc is depicted. In (c), the hydrogen bonding with the amino acids of $A\beta_{1-42}$ 30-mer and cZnPc are shown. The positions of are mentioned beside the 3-letter symbol of amino acids. In (d), the 3-D docking view of $A\beta_{1-42}$ 30-mer and cZnPc is depicted.

Supplemental Figure S4.

A docking simulation was done to understand the interaction of $A\beta_{1-42}$ and cZnPc. In (a), the secondary structure of $A\beta_{1-42}$ 50-mer is shown. In (b), the docking view of $A\beta_{1-42}$ 50-mer and cZnPc is depicted. In (c), the hydrogen bonding with the amino

acids of A$\beta_{1\text{-}42}$ 50-mer and cZnPc are shown. The positions of are mentioned beside the 3-letter symbol of amino acids. In (d), the 3-D docking view of A$\beta_{1\text{-}42}$ 50-mer and cZnPc is depicted.
