# Supplementary material for: A Cationic Zn-Phthalocyanine Turns Alzheimer’s Amyloid β Aggregates into Non-Toxic Oligomers and Inhibits Neurotoxicity in Culture
Source: Int J Mol Sci. 2024 Aug 16;25(16):8931. doi: 10.3390/ijms25168931 (PMC11354870; doi:10.3390/ijms25168931)
Supplement: Supplementary file 1 [file ijms-25-08931-s001.zip › Supplemental figures.pptx]

## Slide 1
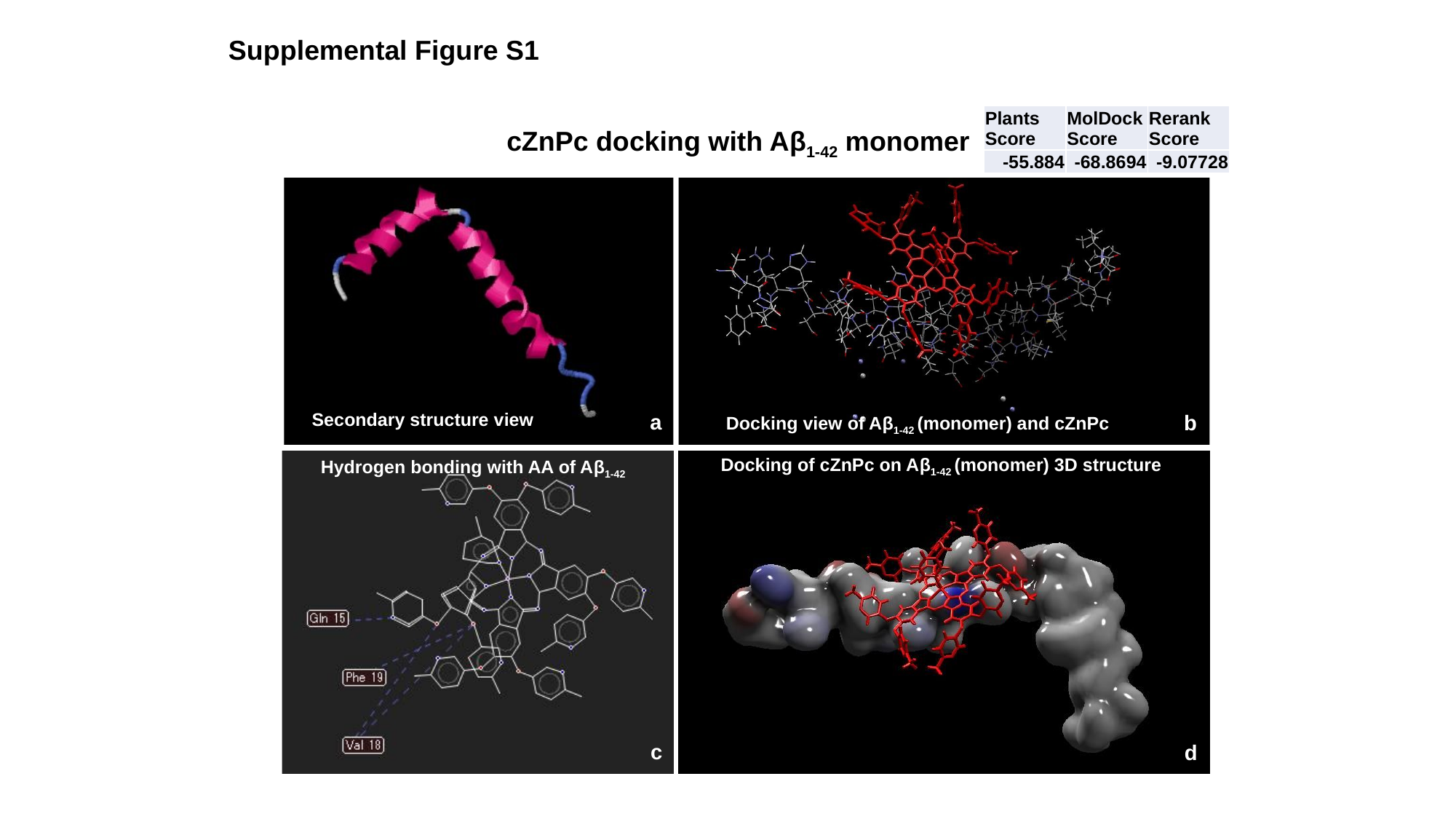

Supplemental Figure S1
| Plants Score | MolDock Score | Rerank Score |
| --- | --- | --- |
| -55.884 | -68.8694 | -9.07728 |
cZnPc docking with Aβ1-42 monomer
Secondary structure view
a
b
Docking view of Aβ1-42 (monomer) and cZnPc
Docking of cZnPc on Aβ1-42 (monomer) 3D structure
Hydrogen bonding with AA of Aβ1-42
c
d

## Slide 2
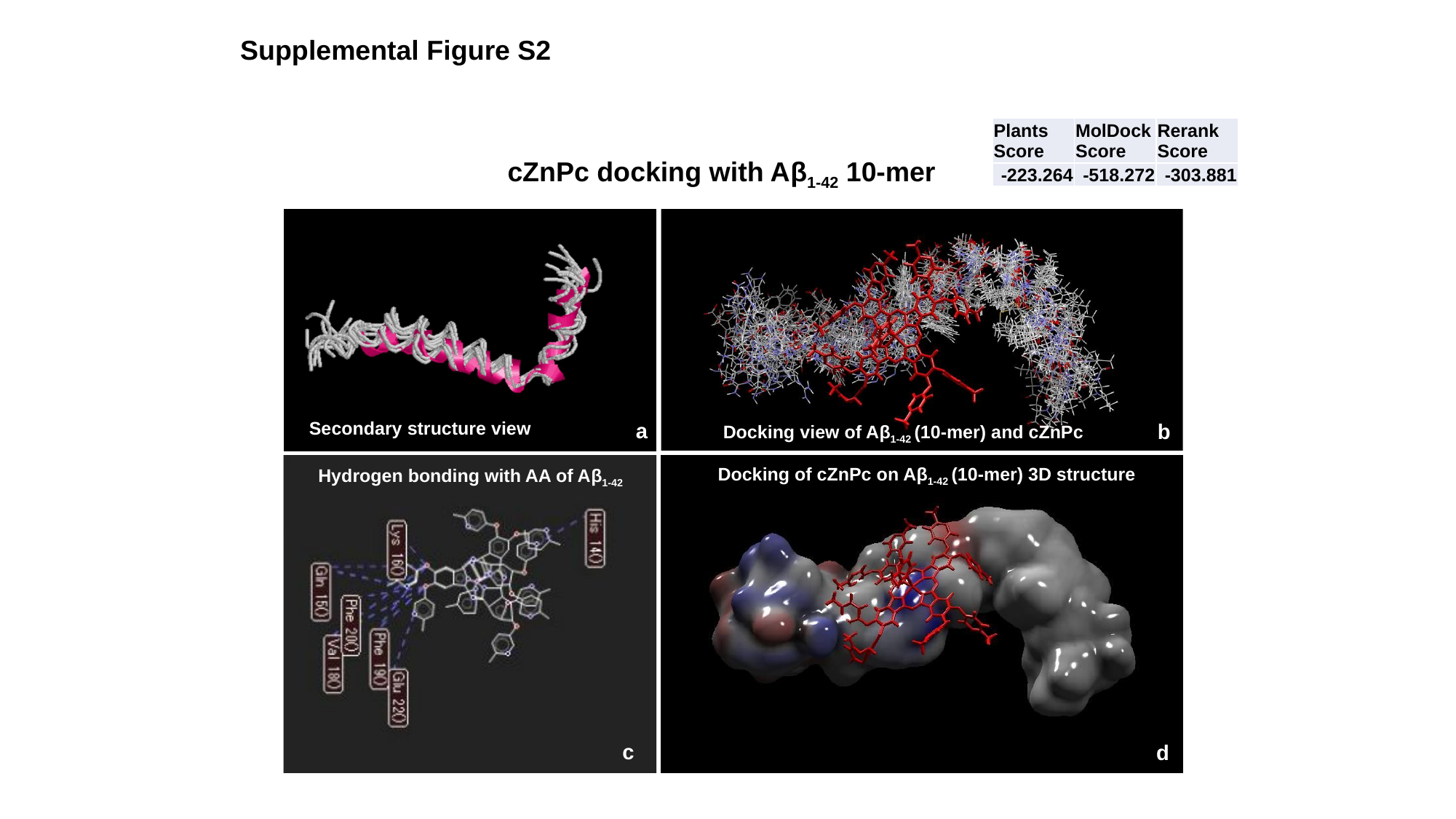

Supplemental Figure S2
| Plants Score | MolDock Score | Rerank Score |
| --- | --- | --- |
| -223.264 | -518.272 | -303.881 |
cZnPc docking with Aβ1-42 10-mer
Secondary structure view
a
b
Docking view of Aβ1-42 (10-mer) and cZnPc
Docking of cZnPc on Aβ1-42 (10-mer) 3D structure
Hydrogen bonding with AA of Aβ1-42
c
d

## Slide 3
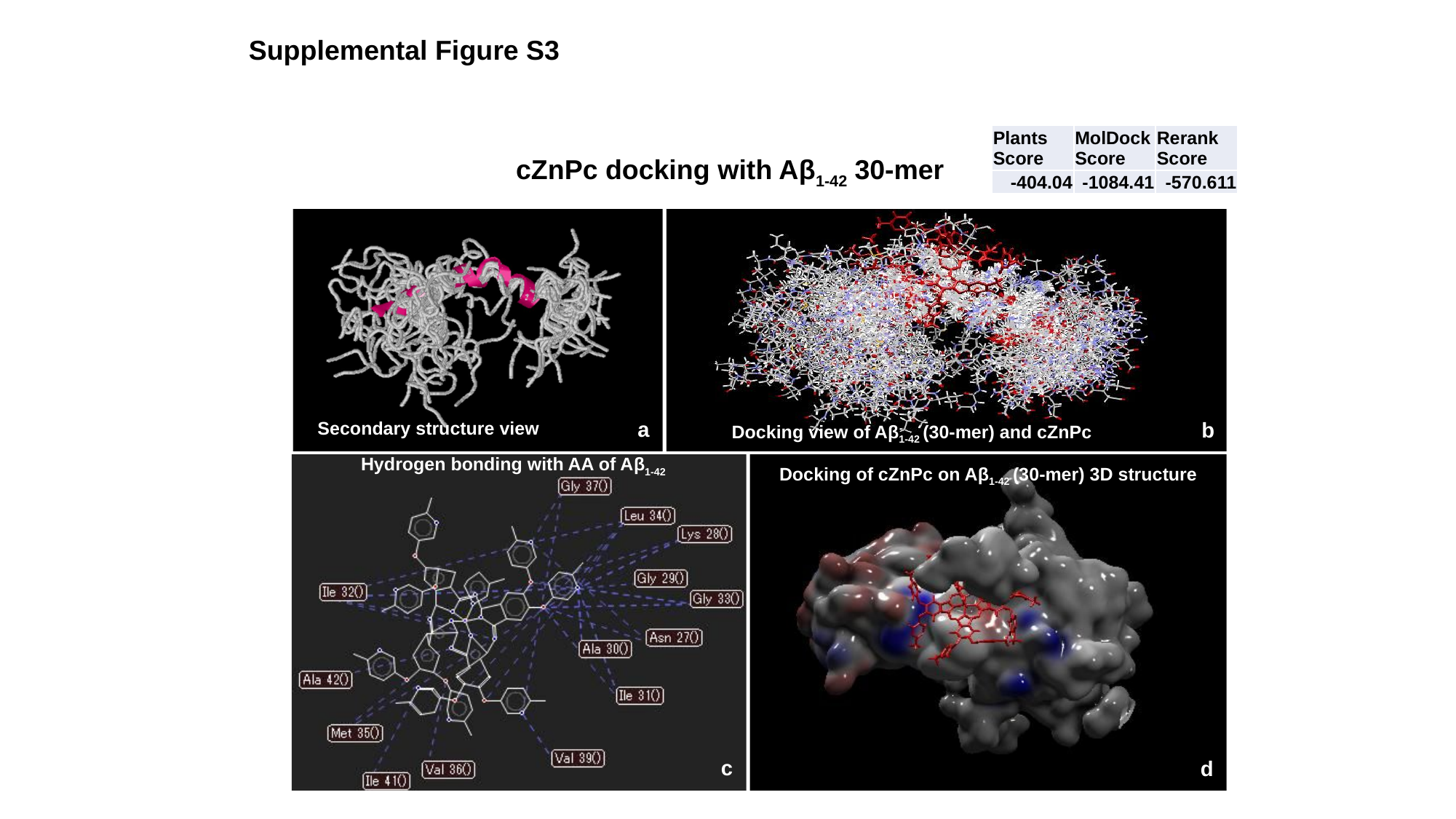

Supplemental Figure S3
| Plants Score | MolDock Score | Rerank Score |
| --- | --- | --- |
| -404.04 | -1084.41 | -570.611 |
cZnPc docking with Aβ1-42 30-mer
a
b
Secondary structure view
Docking view of Aβ1-42 (30-mer) and cZnPc
Hydrogen bonding with AA of Aβ1-42
Docking of cZnPc on Aβ1-42 (30-mer) 3D structure
c
d

## Slide 4
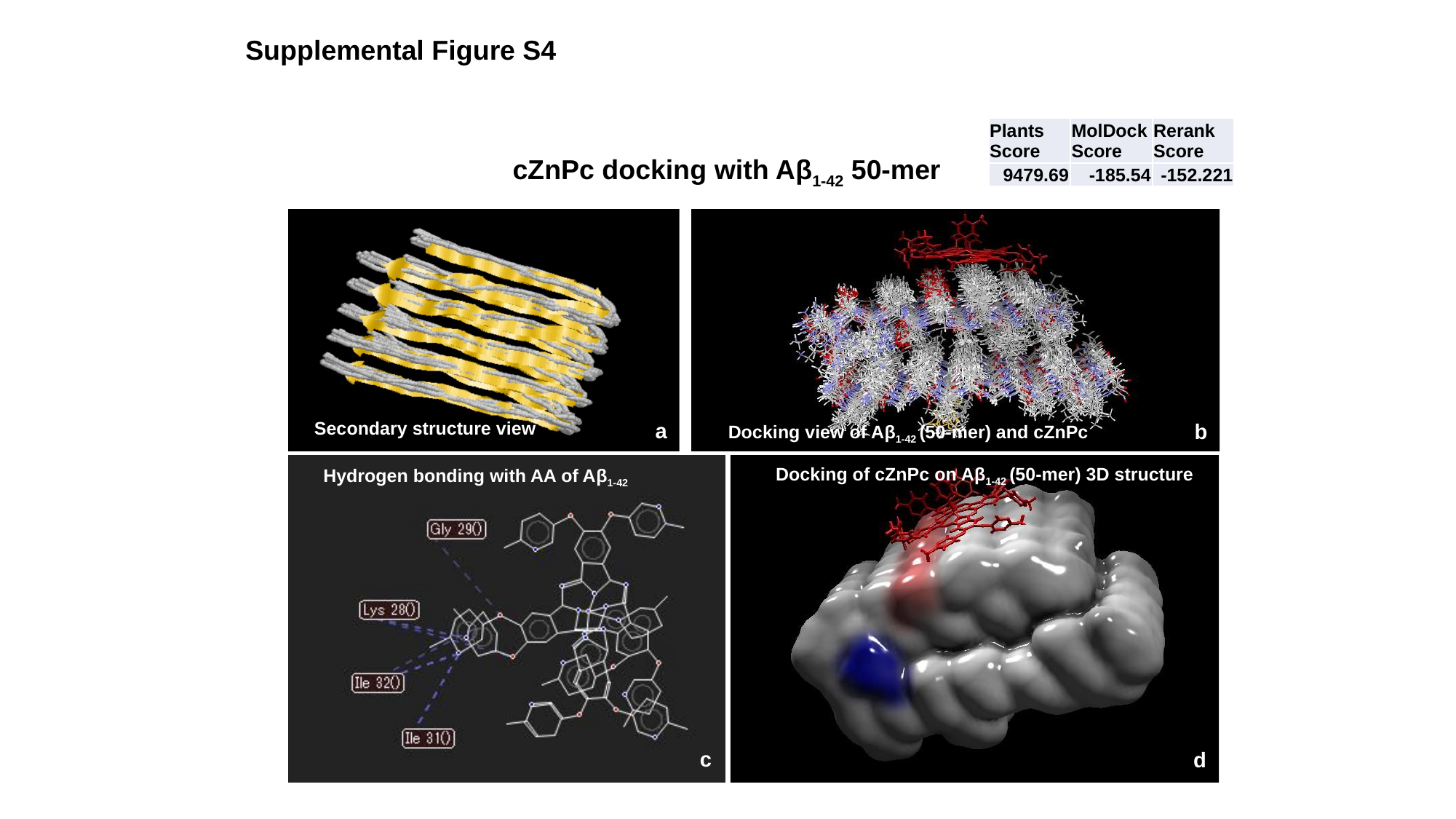

Supplemental Figure S4
| Plants Score | MolDock Score | Rerank Score |
| --- | --- | --- |
| 9479.69 | -185.54 | -152.221 |
cZnPc docking with Aβ1-42 50-mer
Secondary structure view
a
b
Docking view of Aβ1-42 (50-mer) and cZnPc
Docking of cZnPc on Aβ1-42 (50-mer) 3D structure
Hydrogen bonding with AA of Aβ1-42
c
d
